# Occupational prestige, social mobility and the association with lung cancer in men

**DOI:** 10.1186/s12885-016-2432-9

**Published:** 2016-07-07

**Authors:** Thomas Behrens, Isabelle Groß, Jack Siemiatycki, David I. Conway, Ann Olsson, Isabelle Stücker, Florence Guida, Karl-Heinz Jöckel, Hermann Pohlabeln, Wolfgang Ahrens, Irene Brüske, Heinz-Erich Wichmann, Per Gustavsson, Dario Consonni, Franco Merletti, Lorenzo Richiardi, Lorenzo Simonato, Cristina Fortes, Marie-Elise Parent, John McLaughlin, Paul Demers, Maria Teresa Landi, Neil Caporaso, David Zaridze, Neonila Szeszenia-Dabrowska, Peter Rudnai, Jolanta Lissowska, Eleonora Fabianova, Adonina Tardón, John K. Field, Rodica Stanescu Dumitru, Vladimir Bencko, Lenka Foretova, Vladimir Janout, Hans Kromhout, Roel Vermeulen, Paolo Boffetta, Kurt Straif, Joachim Schüz, Jan Hovanec, Benjamin Kendzia, Beate Pesch, Thomas Brüning

**Affiliations:** Institute for Prevention and Occupational Medicine of the German Social Accident Insurance (IPA), Institute of the Ruhr-Universität Bochum, Bürkle-de-la-Camp-Platz 1, 44789 Bochum, Germany; Hospital Research Center (CRCHUM) and School of Public Health, University of Montreal, Montreal, Canada; Dental School, College of Medicine Veterinary and Life Sciences, University of Glasgow, Glasgow, G2 3JZ UK; International Agency for Research on Cancer (IARC), Lyon, France; Institute of Environmental Medicine, Karolinska Institutet, Stockholm, Sweden; Inserm, Centre for Research in Epidemiology and Population Health (CESP), U1018, Environmental Epidemiology of Cancer Team, F-94807 Villejuif, France; University Paris-Sud, UMRS 1018, F-94807 Villejuif, France; Institute for Medical Informatics, Biometry and Epidemiology, University Hospital Essen, Essen, Germany; Leibniz-Institute for Prevention Research and Epidemiology -BIPS GmbH, Bremen, Germany; Institute for Statistics, University Bremen, Bremen, Germany; Institute of Epidemiology I, Helmholtz Zentrum München, Neuherberg, Germany; Unit of Epidemiology, Fondazione IRCCS Ca’ Granda-Ospedale Maggiore Policlinico, Milan, Italy; Department of Medical Sciences, Unit of Cancer Epidemiology, University of Turin, Turin, Italy; Department of Molecular Medicine, Laboratory of Public Health and Population Studies, University of Padova, Padova, Italy; Epidemiology Unit, Istituto Dermopatico dell’Immacolata, Rome, Italy; INRS-Institut Armand-Frappier, Université du Québec, Laval, Québec Canada; Cancer Care Ontario, Occupational Cancer Research Centre, Toronto, Canada; National Cancer Institute, Division of Cancer Epidemiology and Genetics, Bethesda, USA; Institute of Carcinogenesis, Russian Cancer Research Centre, Moscow, Russia; The Nofer Institute of Occupational Medicine, Lodz, Poland; National Centre for Public Health, Budapest, Hungary; The M Sklodowska-Curie Cancer Center and Institute of Oncology, Warsaw, Poland; Regional Authority of Public Health, Preventive Occupational Medicine, Banska Bystrica, Slovakia; Molecular Epidemiology of Cancer Unit, University of Oviedo-Ciber de Epidemiologia, CIBERESP, Oviedo, Spain; Roy Castle Lung Cancer Research Programme, The University of Liverpool Cancer Research Centre, Liverpool, UK; Department of Molecular and Clinical Cancer Medicine, Institute of Translational Medicine, University of Liverpool, Liverpool, UK; National Institute of Public Health, Bucharest, Romania; Institute of Hygiene and Epidemiology, 1st Faculty of Medicine, Charles University, Prague, Czech Republic; Department of Cancer Epidemiology & Genetics, Masaryk Memorial Cancer Institute and Medical Faculty of Masaryk University, Brno, Czech Republic; Faculty of Medicine, Palacky University, Olomouc, Czech Republic; Environmental Epidemiology Division, Institute for Risk Assessment Sciences, Utrecht University, Utrecht, The Netherlands; The Tisch Cancer Institute and Institute for Translational Epidemiology, Icahn School of Medicine at Mount Sinai, New York, USA; Institute of Medical Statistics and Epidemiology, Technical University Munich, Munich, Germany; Faculty of Medicine, University of Ostrava, Ostrava, Czech Republic

**Keywords:** Life course, Occupational history, Social prestige, Socio-economic status, SYNERGY, Transitions

## Abstract

**Background:**

The nature of the association between occupational social prestige, social mobility, and risk of lung cancer remains uncertain. Using data from the international pooled SYNERGY case–control study, we studied the association between lung cancer and the level of time-weighted average occupational social prestige as well as its lifetime trajectory.

**Methods:**

We included 11,433 male cases and 14,147 male control subjects. Each job was translated into an occupational social prestige score by applying Treiman’s Standard International Occupational Prestige Scale (SIOPS). SIOPS scores were categorized as low, medium, and high prestige (reference). We calculated odds ratios (OR) with 95 % confidence intervals (CI), adjusting for study center, age, smoking, ever employment in a job with known lung carcinogen exposure, and education. Trajectories in SIOPS categories from first to last and first to longest job were defined as consistent, downward, or upward. We conducted several subgroup and sensitivity analyses to assess the robustness of our results.

**Results:**

We observed increased lung cancer risk estimates for men with medium (OR = 1.23; 95 % CI 1.13–1.33) and low occupational prestige (OR = 1.44; 95 % CI 1.32–1.57). Although adjustment for smoking and education reduced the associations between occupational prestige and lung cancer, they did not explain the association entirely. Traditional occupational exposures reduced the associations only slightly. We observed small associations with downward prestige trajectories, with ORs of 1.13, 95 % CI 0.88–1.46 for high to low, and 1.24; 95 % CI 1.08–1.41 for medium to low trajectories.

**Conclusions:**

Our results indicate that occupational prestige is independently associated with lung cancer among men.

**Electronic supplementary material:**

The online version of this article (doi:10.1186/s12885-016-2432-9) contains supplementary material, which is available to authorized users.

## Background

Socio-economic position has been observed to be a strong predictor of health inequalities [1]. The incidence of lung cancer varies widely by social class, with the poorest bearing the greatest burden [2]. Although smoking, the most important risk factor in the etiology of lung cancer, explains part of this association, increased lung cancer risk estimates for groups of low socio-economic position persisted in many studies even when controlling for smoking behavior [3–5].

Socio-economic position is a multidimensional construct that may influence health through various mechanisms including occupational, environmental, economic, and behavioral/lifestyle-related exposures, as well as access to health care or health promoting facilities [6]. Theories conceptualizing the mechanisms by which socio-economic position may influence health emphasize structural and interpersonal aspects of different environments, which influence health behaviors and psychological responses to the these environments [7, 8]. Furthermore, the influence of “status inconsistencies” on health have been a focus of socio-epidemiological research: Loss of status control, e.g. incongruity of actual and expected socio-economic position, may impact on a wide range of psychosocial consequences, including chronic stress, mental health/depression, and loss of job control and social support [9], as well as having material circumstances. These factors have also been discussed in relation to cancer risk [10].

In contrast to other measures of socio-economic position [9, 11], Treiman’s Standard International Occupational Prestige Scale (SIOPS) utilizes an internationally comparable scoring system to characterize occupational prestige [12]. Employing precisely defined score values on a metric scale, SIOPS allows for a more detailed assessment of health risks associated with socio-economic position than what is usually available with occupational or social class. However, SIOPS has been rarely employed as a metric of socio-economic position in the epidemiological literature. For example, Schmeisser and co-workers, using SIOPS, identified downward prestige trajectories of occupational prestige during the working life to be an independent risk factor of upper aero-digestive tract cancer [13]. So far, SIOPS has not been analyzed with respect to lung cancer risk.

In addition, the trajectory of occupational prestige over the work life characterizes mobility of a person’s social standing, which permits to consider the development of occupational prestige across the working life instead of prestige at the time of cancer diagnosis [6]. Trajectories of social prestige might entail a wide range of psychosocial variables, incl. work stress, lack of job control, depression, and lack of social support [9]. So far, only few studies have assessed the association between changes of occupational prestige with the risk of cancer, for example [13–15].

SYNERGY (“Pooled Analysis of Case–control Studies on the Joint Effects of Occupational Carcinogens in the Development of Lung Cancer”) has been developed as an international platform into the research of occupational carcinogens and lung cancer. All included case–control studies provided study subjects’ detailed job histories and had solicited detailed information about smoking habits. Smoking information was nearly complete with less than 1 % having missing values [16]. We used this database to study the association between lung cancer and social occupational prestige as well as transitions in life course occupational prestige.

## Methods

The detailed study methods of SYNERGY were described elsewhere [16, 17]. Briefly, SYNERGY is an international collaboration for research into occupational lung cancer. Currently 16 case–control studies from 22 study centers in Italy, France, Germany, the UK, the Czech Republic, Hungary, Poland, Romania, Russia, Slovakia, Spain, Sweden, the Netherlands, Canada, New Zealand, and China are included in this database. Ethical approval for the pooled study was obtained from the IARC Institutional Review Board. National ethics committees approved the local case–control studies. Lung cancer studies were eligible if they obtained a detailed job and smoking history from study subjects.

Interviews were conducted by trained interviewers and 84 % were conducted face-to-face. Most of the included studies used population-based controls (82 %), while some study centers in France (LUCA), Italy (ROME), Spain, the Czech Republic, Hungary, Poland, Slovakia, Romania, Russia, and Canada (TORONTO) obtained control subjects from hospitals (Additional file 1: Table S1). More information about SYNERGY is available on the study’s website on http://synergy.iarc.fr.

Although SIOPS has been shown to be valid in many countries [12], we restricted attention to studies from Europe and Canada for a better comparability of social structures. Because the French PARIS study did not provide information on education and the Dutch MORGEN study did not solicit the time since smoking cessation for former smokers, we excluded these studies. Altogether 12 studies from 13 countries were included in the final analysis. Study subjects or -in the case of deceased subjects- their relatives gave written informed consent to participate in the study.

### Operationalization of occupational prestige

Treiman’s occupational prestige scale assesses the societal socioeconomic hierarchy one associates with a certain job by allocating prestige values to 283 occupations with the minimum value of 14 being assigned to unspecified and unskilled agricultural workers and the maximum (78 points) to physicians and university professors [12]. For this analysis we assigned an occupational prestige score to each occupational period based on a three-digit ISCO-68 (International Standard Classification of Occupations, revision 1968) code. Analyses were restricted to men, because the occupational prestige of women is not directly comparable to men’s, and women tend to have longer periods of economic inactivity in their biography or work part-time more often [18, 19].

The start of occupational activity was determined with the first occupation. Becoming a pensioner was considered the end of a subject’s work history. Missing job periods, were neglected if they lasted two years or less: in these cases, the SIOPS score of the previous job period was assigned. We excluded subjects from the analysis, if job periods with missing information lasted more than two years (*N* = 1,619 (1 % of all job periods)). Moreover, we excluded men with fewer than ten years of lifetime occupational activity (90 subjects).

Job periods starting before the age of 14 or after age 65 years were truncated to ages 14 and 65, respectively. In case of parallel occupations (1,334 job periods from 1,100 subjects), the job with the higher SIOPS score was chosen to determine occupational social prestige.

Intermediate phases of occupational inactivity such as training/education, illness, or unemployment (*N* = 2,279 periods), were assigned a score of 30, as recommended by Treiman [12], which roughly corresponds to the prestige scores of low-skilled manual jobs (such as machinist, plasterer, or vulcanizer) or low clerical work (for example mail distributor, warehouseman). If the occupational prestige was <30 before the period of occupational inactivity, the score value of the preceding job period was assigned to the inactive period. We deleted periods of occupational inactivity before the first occupational activity or after retirement. Periods of imprisonment were assigned a value of 13, which is below Treiman’s minimum value for unskilled agricultural workers.

To assess time-weighted average (TWA) occupational prestige, the products of each prestige score and job period across the entire job history were summed up and then divided by the total duration of the job history. We summarized SIOPS scores according to tertiles of TWA prestige in the control population as low (13- ≤ 35 points, L), medium (>35- ≤ 45 points, M), and high (>45–78 points, H).

Transitions in SIOPS category over the entire job biography were assessed by grouping prestige categories as described above and studying their change from first to last job and from first to longest job, leading to nine different trajectories: consistent (H to H, M to M, and L to L), downward (H to L, H to M, and M to L), and upward (L to H, L to M, and M to H).

### Statistical analysis

To assess lung cancer risk associated with occupational social prestige, we calculated odds ratios (OR) with 95 % confidence intervals (CI) by unconditional logistic regression analysis. “High” prestige was used as reference category. The OR for model 1 was adjusted for study center and age (log-transformed). In model 2, we additionally adjusted for smoking (current smokers, stopped smoking 2–7, 8–15, 16–25 or ≥26 years before interview/diagnosis, other types of tobacco only, non-smokers, and cumulative tobacco consumption (log(pack-years + 1)). Current smokers included smokers who had quit ≤1 year before interview/diagnosis. We defined non-smokers as never smokers plus subjects with a smoking history of <1 pack-year. Model 3 added ever employment in occupations with an established lung cancer risk (“List A” job, yes/no), including, among others, jobs in metal production and processing, construction, mining, the chemical industry, asbestos production, etc. [20, 21]. The fully adjusted model 4 furthermore included education (no formal/some primary education (<6 years), primary/some secondary education (6–9 years), secondary education/some college (10–13 years), university/college degree) [22].

To visualize the functional form of the adjusted dose–response association between TWA occupational prestige and lung cancer for model 4, we calculated restricted cubic spline functions and associated 95 % CI with four knots located at the 5th, 25th, 75th, and 95th percentiles. Median TWA occupational prestige in the control population (40 points) was chosen as reference.

We used random-effect meta-regression models to pool ORs of individual studies. Statistical analyses were carried out with SAS, version 9.2 (SAS Institute Inc., Cary, NC) and Comprehensive Meta-Analysis Version 2.2.027 software (Biostat, Englewood, NJ).

### Subgroup and sensitivity analyses

We conducted several subgroup analyses to assess the robustness of our results. We stratified analyses by study region (eastern (Czech Republic, Hungary, Poland, Romania, Russia, Slowakia), southern (Italy, Spain), northern Europe (Germany, Sweden, France, UK), and Canada), smoking status, major histological subtype of lung cancer, educational level, blue collar worker status (defined as an ISCO-68 first digit of 7, 8, or 9), and employment in a “List A” job.

We conducted sensitivity analyses leaving out each study. Further, we varied class borders for occupational prestige category using three equidistant categories each comprising 22 occupational prestige codes: low (13–34 points), medium (35–56 points) and high (57–78 points), as well as an equal number of occupations (three-digit ISCO-codes) for each category (13–33, 34–45, and 46–78 points, respectively) [13]. We also used a SIOPS-classification applying five occupational groups which were constructed along the line of manual/non manual job and perceived autonomy of action [23], as shown in Additional file 1: Table S4.

## Results

The final data set included 11,433 male cases and 14,147 male control subjects. Median age was 63 years. Most subjects were smokers or former smokers. Educational levels were rather low: About 46 % of subjects had only 6–9 years of school education, and 16 % had fewer than six years of schooling (Table 1).

**Table 1:** Study characteristics by case–control status

		Cases (*n* = 11,433)		Controls (*n* = 14,147)	
		N	%	N	%
Age category	20- <40 years	109	1.0	199	1.4
	40- <50 years	934	8.2	1,287	9.1
	50- <60 years	3,040	26.6	3,597	25.4
	60- <70 years	4,657	40.7	5,809	41.1
	70- <80 years	2,616	22.9	3,210	22.7
	≥80 years	77	0.7	45	0.3
Age [years]	Median (interquartile range)	63 (56–69)		63 (56–69)	
Smoking status	Non-smoker	279	2.4	3,506	24.8
	Former smoker	3,957	34.6	6,321	44.7
	Current smoker	7,051	61.7	3,950	27.9
	Other types of tobacco only	146	1.3	370	2.6
Cumulative tobacco consumption [pack-years] in former and current smokers	Median (interquartile range)	39 (27–53)		23 (11–38)	
Educational level	<6 years	2,210	19.3	1,857	13.1
	6–9 years	5,689	49.8	5,994	42.4
	10–13 years	2,295	20.1	3,718	26.3
	University degree	1,239	10.8	2,578	18.2
List A occupation	Never	9,808	85.8	12,878	91.0
	Ever	1,625	14.2	1,269	9.0
Blue/White collar worker	Blue collar	6,284	55.0	5,828	41.2
	White collar	3,803	33.3	6,751	47.7
	Mixed blue/white collar	1,346	11.8	1,568	11.1
Last residence	Urban (≥10,000 inhabit.)	7,389	64.6	9,004	63.6
	Rural (<10,000 inhabit.)	1,816	15.9	1,849	13.1
	Missing	2,228	19.5	3,294	23.3
Time-weighted average occupational social prestige	High (>45- 78 points)	2,215	19.4	4,592	32.5
	Medium (>35- ≤45 points)	3,980	34.8	4,854	34.3
	Low (13- ≤35 points)	5,238	45.8	4,701	33.2
Histological lung cancer subtype	Squamous cell cancer	4,875	42.6		
	Small cell lung cancer	1,843	16.1		
	Adenocarcinoma	2,818	24.6		
	Other or mixed	1,825	16.0		
	Missing	72	0.6		

The vast majority of cases with <9 years of schooling had low prestige occupations (86.2 % among cases and 79.1 % among control subjects), whereas almost all subjects with university degrees were in the high occupational prestige category. Subjects with low prestige were more likely to have ever smoked than subjects with high occupational prestige (96.3 vs. 79 %) (results not shown).

### Associations between lung cancer and occupational prestige

**Fig. 1:**
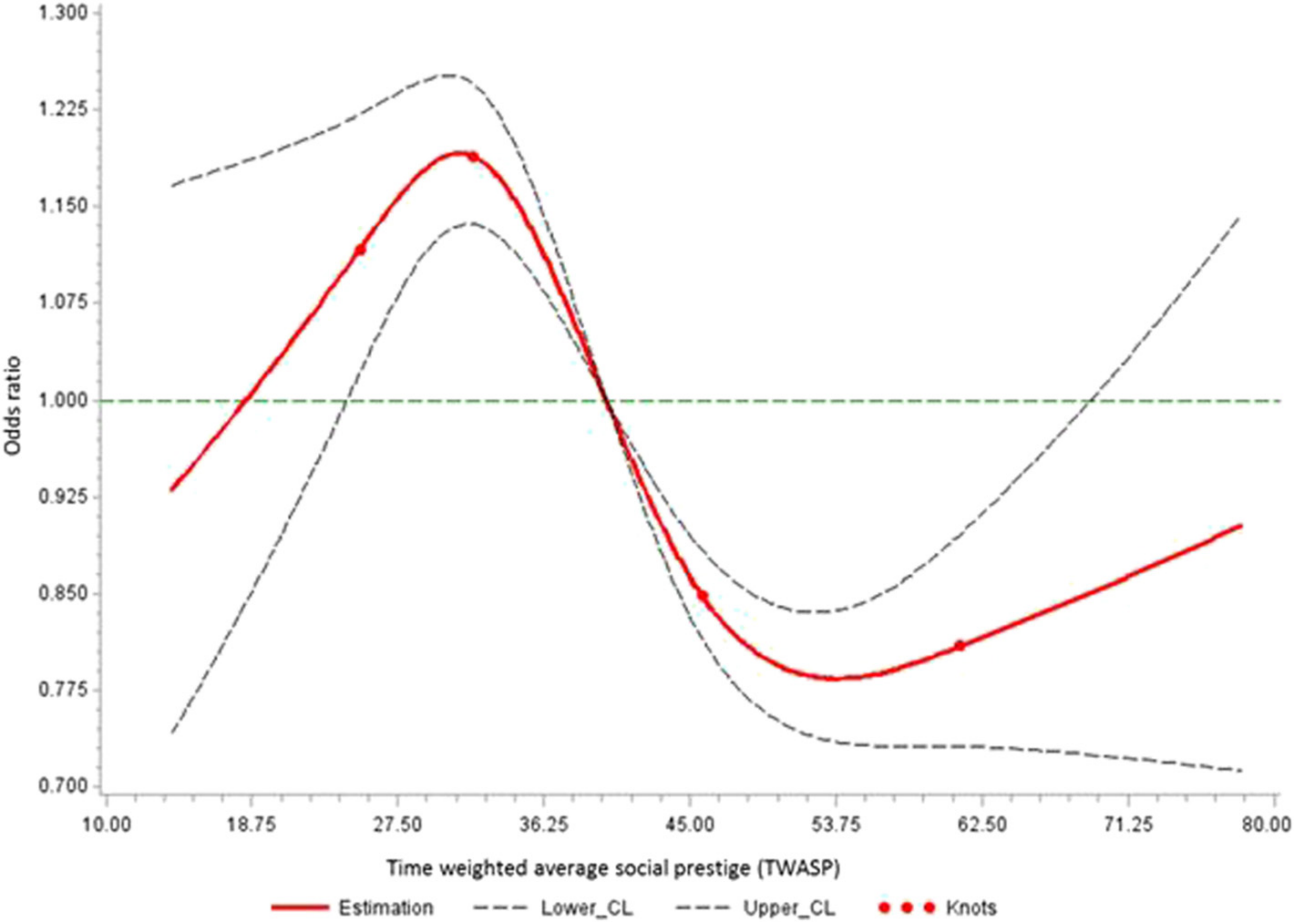
Estimated exposure-response association for time-weighted average occupational social prestige and lung cancer risk with restricted cubic spline function with 4 knots located at the 5th, 25th, 75th and 95th percentiles of the distribution of TWASP adjusted for study center, log(age), smoking status with time since quitting, log(pack-years + 1), ever working in List A occupation and education (model 4). Reference value is 40, the median of time-weighted average social prestige in the control population. The dashed lines are the lower and upper 95 % confidence limits. Tests for overall association and also for non-linear association were significant with p-values <0.0001

Table 2 displays the ORs for lung cancer and TWA occupational prestige for four models entailing different covariates. In models 1 there were strong effects of occupational prestige on lung cancer risk. Adjustment for smoking and education had an attenuating effect, whereas adjustment for exposure to List A jobs had little impact (<10 %) on the association. The general pattern of results seen for all lung cancers in Table 2 was also seen for the main histologic types, squamous cell and small cell cancer, but not clearly for adenocarcinomas. Estimated dose–response associations for TWA occupational prestige using cubic spline functions are shown in Fig. 1, indicating a statistically significant overall trend (*p* < 0.0001) for the non-linear association.

**Table 2:** Odds ratios (OR) with 95 % confidence intervals (CI) between lung cancer and categories of time-weighted average occupational social prestige for all lung cancers combined and major histological subtypes of lung cancer

Type of lung cancer/Social prestige category ^a^	Cases [N]	Controls [N]	OR1 ^b^ (95 % CI)	OR2 ^c^ (95 % CI)	OR3 ^d^ (95 % CI)	OR4 ^e^ (95 % CI)
All lung cancers						
High	2,215	4,592	1.0	1.0	1.0	1.0
Medium	3,980	4,854	1.67 (1.56–1.78)	1.39 (1.29–1.50)	1.37 (1.27–1.47)	1.23 (1.13–1.33)
Low	5,238	4,701	2.32 (2.17–2.48)	1.74 (1.61–1.87)	1.68 (1.55–1.81)	1.44 (1.32–1.57)
Squamous cell carcinoma						
High	812	4,592	1.0	1.0	1.0	1.0
Medium	1,705	4,854	1.93 (1.76–2.12)	1.56 (1.41–1.73)	1.54 (1.39–1.71)	1.29 (1.15–1.45)
Low	2,358	4,701	2.85 (2.60–3.12)	2.08 (1.88–2.30)	2.03 (1.83–2.25)	1.58 (1.40–1.78)
Small cell carcinoma						
High	324	4,592	1.0	1.0	1.0	1.0
Medium	638	4,854	1.89 (1.64–2.18)	1.48 (1.27–1.72)	1.44 (1.24–1.68)	1.29 (1.10–1.53)
Low	881	4,701	2.78 (2.42–3.19)	1.94 (1.67–2.24)	1.86 (1.60–2.16)	1.62 (1.37–1.92)
Adenocarcinoma						
High	690	4,592	1.0	1.0	1.0	1.0
Medium	963	4,854	1.27 (1.14–1.42)	1.10 (0.98–1.24)	1.08 (0.96–1.21)	1.01 (0.89–1.15)
Low	1,165	4,701	1.64 (1.47–1.82)	1.28 (1.14–1.43)	1.22 (1.09–1.37)	1.13 (0.99–1.29)

a Categories for social prestige scores according to tertiles among control subjects: Low = 13- ≤ 35, Medium = >35- ≤ 45, and High = >45-78 points

b Odds ratios for model 1 are adjusted for study center and log(age)

c Odds ratios for model 2 are additionally adjusted for smoking status with time since quitting (2–7, 8–15, 16–25 or ≥ 26 years before interview/diagnosis, other types of tobacco only, non-smokers), and log(pack-years + 1)

d Odds ratios for model 3 are additionally adjusted for ever working in “List A” occupation

e Odds ratios for model 4 are additionally adjusted for highest education

When we conducted a meta-analysis of low vs. high prestige in the different studies, there was statistically significant heterogeneity among studies, with an I^2^ of 61 %. The studies showing the highest ORs between low occupational prestige and lung cancer were from Germany, Canada, France, and some studies from Eastern Europe (Additional file 1: Figure S1).

### Time course of occupational prestige

Risk estimates for downward trajectories to low social occupational prestige were elevated in the crude model adjusting only for study center and age. Further adjustment for smoking diminished the associations. Adjustment for List A occupation had only a small effect on the risk estimates. After further adjustment for education the associations were slightly increased, e.g. for a change from high to low prestige from first to last occupation OR = 1.13 (95 % CI 0.88–1.46), or from medium to low prestige of OR = 1.24 (95 % CI 1.08–1.41), respectively. Increased risk estimates were observed for consistently low or medium trajectories of prestige. In contrast, upward trajectories (low to high or medium to high) were rather associated with a decrease in lung cancer risk estimates (Table 3). Stratification by educational level yielded heterogeneous results, and we did not identify a clear education-dependent pattern of increased ORs as seen in the analysis of categories of occupational prestige. For example, medium to low trajectories of occupational social prestige (first to last job) were associated with an increased risk only in subjects with low educational levels <10 years, whereas for trajectories of high to low prestige increased estimates were only implied among subjects with medium educational level or a university degree (not shown). Ever being unemployed for more than one year was not associated with an increased lung cancer risk in our data (OR = 1.04; 95 % CI 0.95–1.15).

**Table 3:** Odds ratios (OR) with 95 % confidence intervals (CI) between lung cancer and transition in time-weighted average occupational social prestige categories for first occupation to last occupation and first occupation to longest occupation

Transitions in social prestige categories ^a^		Cases [N]	Controls [N]	OR1 ^b^ (95 % CI)	OR2 ^c^ (95 % CI)	OR3 ^d^ (95 % CI)	OR4 ^e^ (95 % CI)
Change in social prestige from first to last occupation							
Consistent	H to H	1,088	2,333	1.0	1.0	1.0	1.0
	M to M	1,796	2,106	1.71 (1.55–1.88)	1.40 (1.25–1.56)	1.37 (1.23–1.53)	1.20 (1.06–1.35)
	L to L	3,960	3,567	2.29 (2.10–2.50)	1.63 (1.48–1.80)	1.57 (1.42–1.74)	1.31 (1.17–1.45)
Downward	H to L	168	210	1.70 (1.37–2.11)	1.33 (1.03–1.71)	1.28 (0.99–1.65)	1.13 (0.88–1.46)
	H to M	144	244	1.20 (0.96–1.49)	1.03 (0.81–1.32)	1.03 (0.80–1.32)	0.95 (0.74–1.22)
	M to L	1,386	1,303	2.08 (1.87–2.31)	1.52 (1.34–1.71)	1.46 (1.29–1.65)	1.24 (1.08–1.41)
Upward	M to H	963	1,781	1.04 (0.93–1.16)	0.94 (0.83–1.07)	0.93 (0.82–1.05)	0.87 (0.77–0.99)
	L to H	832	1,451	1.15 (1.03–1.29)	0.94 (0.83–1.07)	0.92 (0.81–1.05)	0.83 (0.73–0.95)
	L to M	1,096	1,152	1.88 (1.68–2.10)	1.45 (1.28–1.65)	1.40 (1.23–1.59)	1.19 (1.04–1.36)
Change in social prestige from first occupation to longest occupation							
Consistent	H to H	1,155	2,417	1.0	1.0	1.0	1.0
	M to M	2,154	2,497	1.69 (1.54–1.85)	1.38 (1.24–1.53)	1.35 (1.21–1.50)	1.17 (1.05–1.31)
	L to L	4,108	3,799	2.18 (2.0–2.38)	1.57 (1.42–1.73)	1.51 (1.37–1.66)	1.26 (1.13–1.40)
Downward	H to L	123	155	1.63 (1.27–2.10)	1.22 (0.91–1.62)	1.17 (0.88–1.56)	1.02 (0.77–1.37)
	H to M	122	215	1.10 (0.87–1.39)	0.93 (0.72–1.21)	0.93 (0.71–1.21)	0.85 (0.65–1.11)
	M to L	1,157	1,092	2.0 (1.79–2.23)	1.43 (1.27–1.63)	1.38 (1.22–1.57)	1.16 (1.01–1.32)
Upward	M to H	834	1,601	0.97 (0.86–1.08)	0.90 (0.80–1.02)	0.89 (0.79–1.01)	0.84 (0.74–0.96)
	L to H	724	1,260	1.11 (0.99–1.25)	0.92 (0.81–1.05)	0.90 (0.79–1.03)	0.82 (0.71–0.94)
	L to M	1,056	1,111	1.83 (1.63–2.04)	1.40 (1.23–1.59)	1.35 (1.18–1.53)	1.14 (0.99–1.31)

^a^ Categories for occupational social prestige scores according to tertiles among control subjects: Low (L) = 13- ≤ 35, Medium (M) = >35- ≤ 45, and High (H) = >45–78 points

^b^ Odds ratios for model 1 are adjusted for study center and log(age)

^c^ Odds ratios for model 2 are additionally adjusted for smoking status with time since quitting (2–7, 8–15, 16–25 or ≥26 years before interview/diagnosis, other types of tobacco only, non-smokers), and log(pack-years + 1)

^d^ Odds ratios for model 3 are additionally adjusted for ever working in “List A” occupation

^e^ Odds ratios for model 4 are additionally adjusted for highest education

**Fig. 2:**
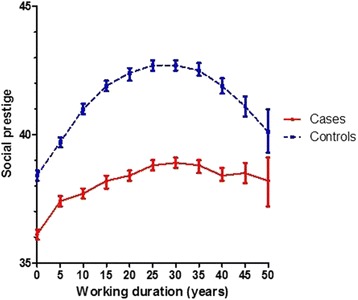
Unadjusted time course of mean occupational social prestige with 95 % confidence intervals for working durations from 0 to 50 years (by intervals of 5 years) for cases and controls (class limits based on tertiles of the distribution of TWA-prestige among controls)

**Fig. 3:**
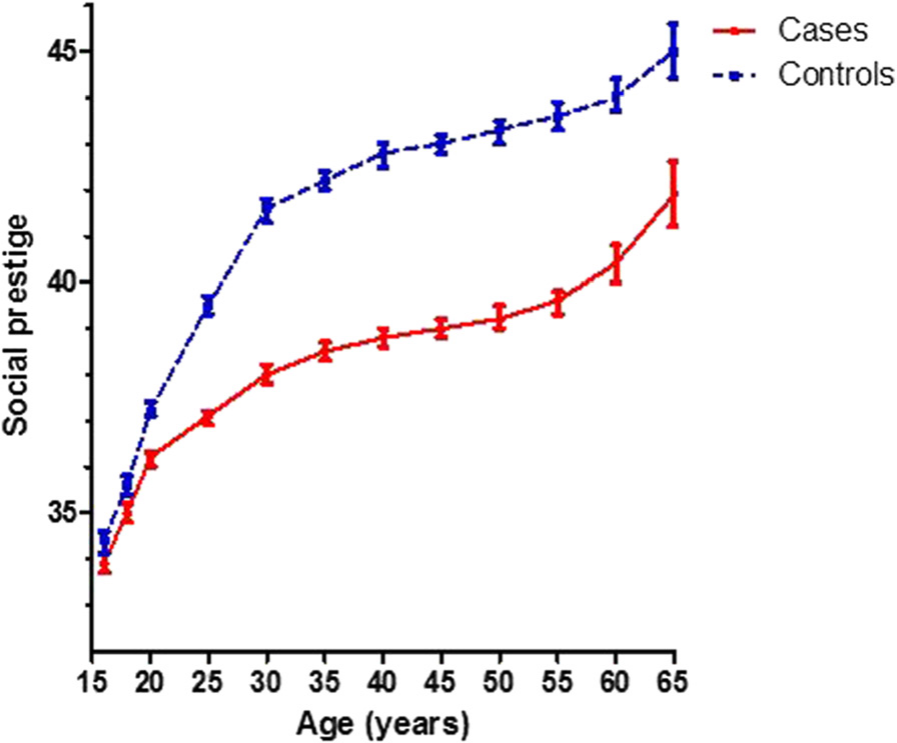
Unadjusted time course of mean occupational social prestige with 95 % confidence intervals for age (by intervals of 5 years) for cases and controls (class limits based on tertiles of the distribution of TWA-prestige among controls)

Comparing the time course of mean occupational prestige according to work duration (Fig. 2) and age (Fig. 3) between cases and controls revealed that cases consistently had lower prestige scores than control subjects. The difference slightly increased until age 20–30 years and remained stable thereafter. This tendency did not depend on the first job’s social occupational prestige (Additional file 1: Figures S2-S7).

### Subgroup and sensitivity analyses

The overall pattern of excess risk with low occupational prestige held within strata of smoking characteristics. Even among non-smokers, there was an elevated risk among those with low occupational prestige compared to those with high prestige. East European countries showed slightly lower ORs as compared to Northern Europe and Canada. In southern European studies the OR was only slightly elevated for the low prestige category (Table 4).

**Table 4:** Odds ratios between lung cancer and categories of time-weighted average occupational social prestige in various subgroups of the study population

Subpopulation	Occupational prestige	Cases [N]	Controls [N]	OR (95 % CI)
Study Region				
Northern Europe ^a^	High	1,244	3,000	1.0
	Medium	2,206	2,899	1.23 (1.11–1.38)
	Low	2,876	2,629	1.60 (1.43–1.80)
Southern Europe ^a^	High	452	710	1.0
	Medium	943	1,104	1.08 (0.89–1.30)
	Low	1,183	1,036	1.19 (0.96–1.46)
East Europe ^a^	High	377	553	1.0
	Medium	677	628	1.27 (1.02–1.57)
	Low	871	722	1.24 (0.98–1.56)
Canada ^a^	High	142	329	1.0
	Medium	154	223	1.37 (0.96–1.95)
	Low	308	314	1.53 (1.06–2.21)
Smoking status				
Current smokers ^b^	High	1,217	947	1.0
	Medium	2,388	1,424	1.12 (0.99–1.27)
	Low	3,446	1,579	1.38 (1.22–1.58)
Former smokers ^b^	High	886	2,121	1.0
	Medium	1,454	2,169	1.31 (1.15–1.48)
	Low	1,617	2,031	1.42 (1.24–1.62)
Non-smokers ^b^	High	81	1,366	1.0
	Medium	92	1,152	1.27 (0.90–1.81)
	Low	106	988	1.64 (1.13–2.37)
Educational level				
<6 years ^c^	High	97	143	1.0
	Medium	643	606	1.57 (1.13–2.18)
	Low	1,470	1,108	1.70 (1.22–2.37)
6–9 years ^c^	High	541	957	1.0
	Medium	2,105	2,426	1.35 (1.18–1.55)
	Low	3,043	2,611	1.56 (1.35–1.80)
10–13 years ^c^	High	761	1,518	1.0
	Medium	944	1,390	1.20 (1.05–1.38)
	Low	590	810	1.18 (0.98–1.42)
University/college degree ^c^	High	816	1,974	1.0
	Medium	288	432	1.08 (0.88–1.32)
	Low	135	172	0.97 (0.68–1.36)
Occupation				
Never “List A” job ^d^	High	2,128	4,458	1.0
	Medium	3,428	4,412	1.21 (1.11–1.32)
	Low	4,252	4,008	1.47 (1.34–1.61)
Ever “List A” job ^d^	High	87	134	1.0
	Medium	552	442	1.53 (1.08–2.17)
	Low	986	693	1.63 (1.15–2.32)
White collar job ^a^	High	1,833	3,989	1.0
	Medium	1,084	1,712	1.09 (0.97–1.22)
	Low	886	1,050	1.30 (1.13–1.50)
Blue collar job ^a^	High	186	277	1.0
	Medium	2,370	2,457	1.24 (0.99–1.55)
	Low	3,728	3,094	1.43 (1.14–1.79)
Mixed blue/white collar ^a^	High	196	326	1.0
	Medium	526	685	1.08 (0.85–1.39)
	Low	624	557	1.38 (1.06–1.79)

^a^ ORs adjusted for study center, log(age), smoking status with time since quitting (2–7, 8–15, 16–25 or ≥ 26 years before interview/diagnosis, other types of tobacco only, non-smokers), and log(pack-years + 1), ever working in “List A” occupation, and highest school education

^b^ ORs adjusted for study center, log(age), ever working in “List A” occupation, and highest school education, pack-years and other types of tobacco only

^c^ Model as in (a) without adjustment for educational level

^d^ Model as in (a) without adjustment for “List A” job

When we stratified analyses by educational level, the highest ORs between occupational prestige and lung cancer were observed for subjects with medium and low occupational social prestige and low school education: <6 years OR = 1.57; 95 % CI 1.13–2.18 and OR = 1.70; 95 % CI 1.22–2.37 and for education of 6–9 years OR = 1.35; 95 % CI 1.18–1.55 and OR = 1.56; 95 % CI 1.35–1.80, respectively. We observed increased risk estimates in subjects with 10–13 years of school education, whereas no increase in lung cancer risk was seen in subjects with a university degree (Table 4). The model including an interaction term of TWASP tertiles and educational level yielded a statistically significant interaction term (*p* = 0.027) (not shown).

Stratification by white and blue collar job demonstrated higher risk estimates for low prestige blue collar workers and an analogous phenomenon was observed among white collar workers, and among subgroups of workers working in List A jobs, as well as those not working in List A jobs (Table 4). Analyses leaving out each study one by one did not indicate a dominant influence by a single study (for results excluding study regions see Additional file 1: Table S5).

Varying the definition of class borders for TWA occupational prestige categories did not change results much (Additional file 1: Table S3). The analysis of five occupational classes according to perceived job autonomy indicated that ORs were greater when job autonomy was lowest (Additional file 1: Table S4). Male manual workers with low and very low autonomy showed the highest risk estimates in the fully adjusted model, however the social gradient was less strong as compared to the analyses using tertiles of TWA prestige.

## Discussion

In this comprehensive analysis of more than 11,000 male cases and 14,000 control subjects we observed a social gradient of occupational prestige with lung cancer risk. The associations were not fully explained by occupational exposures or smoking habits and persisted when we restricted our analysis to non-smokers. Analyses of transitions of occupational prestige indicated the strongest associations for consistently low trajectories during the work life.

One strength of this study is the detailed assessment of smoking behavior and the large number of non-smoking cases.

Further strengths of our analysis are that we solicited the study subjects’ full work history, which enabled us to consider occupational prestige across the working life instead at the time of cancer diagnosis only. Changes in socio-economic position over time (and associated loss of income, social support, and social standing) may have profound implications for later health, which we addressed in our analysis of trajectories in occupational prestige.

Limitations include that grouping job titles according to their occupational prestige may not reflect a profession’s real prestige in a society [24], which also may differ according to socio-cultural background in different countries. However, occupational prestige as assessed with SIOPS was found to be internationally comparable and has been validated with ISCO data from surveys in more than 50 countries [12]. We cannot rule out that study subjects in some countries may have inflated their job titles to infer greater prestige. Because the job history was solicited to assess occupational exposures to lung carcinogens and translated to ISCO codes by independent coders, we believe this bias to be rather unlikely though.

A single occupation’s prestige may also change over time, in particular in the context of profound societal changes, such as industrialization or change of the political system. Interestingly, in the SIOPS data, which were collected within a 20-year period and in politically diverse countries such as the U.S.A., Belgium, Iraq, or the former U.S.S.R., the ranking of jobs according to their social prestige was independent from country or time of survey [12]. Compared to other measures of social status that incorporate income and education, occupation appears to be less affected by temporal changes: Educational levels have increased over time in many countries, whereas incomes have stagnated or even decreased. Occupation, which also encompasses aspects of education and income may therefore be considered a rather stable indicator for socioeconomic position [23].

Another limitation is that we only considered occupation in a List A job to assess the influence of occupational exposures to known lung carcinogens on the association between occupational prestige and lung cancer risk. However, our results are in line with the EPIC study cohort which identified only a small influence of occupational exposures to asbestos, polycyclic aromatic hydrocarbons, and heavy metals on educational inequalities in lung cancer incidence [25].

Further limitations include that we could not directly consider other indicators of socio-economic position (such as income or ethnicity), which may have independent effects on health inequalities [9, 26]. We were not able to consider early life or other contextual influences (such as family’s socio-economic position or neighborhood characteristics) either. These factors may influence vulnerability to adult health risks during the life course [27, 28], although their influence on lung cancer risk appears to be rather small [29]. Interestingly, when comparing the time course of occupational social prestige during the work life, we observed consistently lower prestige score among cases occurring at an early age or early in the work life (Figs. 2 and 3), which implies influences on lung cancer risk that may work before the start of an occupational career.

For this analysis we used the most detailed information with respect to smoking habits to avoid residual confounding by smoking status to a large extent, as previously recommended in a SYNERGY sub-study [30]. We confirmed that smoking was a major confounder in our analysis, but a positive association of low occupational prestige with lung cancer persisted, when we restricted the analysis to non-smoking subjects. This pattern was also seen in a large cohort of more than 22,000 Swedish individuals from the city of Malmö [31]. Because we classified subjects with a smoking-history of <1 pack-year as non-smokers, residual confounding by smoking cannot be completely ruled out. We observed stronger effects for squamous cell and small cell lung cancer, whereas risk estimates for adenocarcinoma of the lung were only slightly increased in the fully adjusted model. This observation may point towards residual confounding by smoking, because adenocarcinoma is the histological subtype of lung cancer showing the weakest association with smoking behavior [17].

We cannot rule out either that reporting of smoking behavior was biased due to differential recall between subjects with high and low occupational prestige. Previous research has demonstrated good agreement between self-reported smoking behavior and serum cotinine levels though, and the difference by socio-economic characteristics was marginal (3 % of blue collar workers vs. 1 % of white collar workers reporting no exposure to tobacco smoke, but were classified as smokers according to their cotinine levels) [32].

In addition, the pooled SYNERGY study population consists of countries that are in different phases of the smoking epidemic with changing relationship on social classes and cigarette smoking. This applies in particular to southern European countries, which are in an earlier stage of the smoking epidemic than countries in the north [33]. This may explain why the association between social occupational prestige and lung cancer in SYNERGY was weaker in these regions. Cultural factors in socio-economic development and history are considered to ameliorate differences in lifestyle independently from social status (or social prestige) [3, 34, 35]. In addition, different schooling systems (e.g. mandatory school education of at least 10 years in most former Communist countries) could have also contributed to the heterogeneous results observed in the different SYNERGY regions (Additional file 1: Figure S1).

Education was shown to be a major confounder in our analysis. When choosing a model adjusting for education, we cannot rule out over-adjustment due to the correlation of occupational prestige and educational level (Cramer’s V = 0.39) which could have biased our risk estimates towards unity. Correlations differed only slightly between study regions, ranging from Cramer’s V 0.38 in East Europe to 0.48 in Southern Europe. In the stratified analysis according to education the association between lower occupational prestige and lung cancer risk estimates diminished with increasing educational level. Study subjects holding a university degree, which reflects the starting point for a professional career encompassing jobs with high occupational prestige, did not show any association of lung cancer with occupational prestige. However, the strong influence of education in the stratified results may also be seen as an indicator that adverse social circumstances are determined by behavioral or environmental factors early in life which may accumulate over the life course [36].

Few studies so far have studied the influence of social mobility on the risk of cancer. As earlier research suggested, loss of self-control is one of the pivotal elements in the manifestation of stress and, and thus occupational careers with undesired downward social mobility may serve as important reference points for chronic life strain [37]. A French research group investigated the effect of occupational position on lung cancer risk at three different career points in a government-owned electricity company. At all career points, the employment in the lowest category was associated with an increased lung cancer risk as compared to the highest category. However, risk estimates between the three career points differed and were highest at the time of diagnosis, emphasizing the need to assess social change as influencing factor on the association with cancer [14]. Another study similar to the one presented here found that upper aero-digestive tract cancer was associated with downward drift of occupational prestige during the working life [13]. In our analysis a possible influence of social distress on lung cancer was implied by our findings of slightly increased risk estimates with downward trajectories of occupational prestige, and decreased associations with upward drift during the work life. Together with our observation of a positive association with last, but not first job prestige after adjusting for education (Additional file 1: Table S2) this may suggest a sustainable beneficial effect of high prestige in early life, whereas high prestige in later life may exert a positive effect on cancer risk with a shorter latency.

## Conclusions

In summary, we found that low occupational prestige in men was associated with lung cancer independent of smoking habits and occupational exposures. Lung cancer cases had lower social prestige scores occurring early in life, and this difference remained stable during the entire work life. In contrast, associations for downward trajectories with lung cancer appeared to be less relevant and were mostly explained by smoking behavior and education. While smoking cessation is clearly the most important objective for primary prevention of lung cancer, it remains pertinent to understand the potential contributions and mechanisms of other factors, such as occupational prestige.

## Abbreviations

CI, confidence interval; H, high; ISCO-68, international standard classification of occupations, rev. 1968; L, low; M, medium; OR, odds ratio; SIOPS, standard international occupational prestige scale; SYNERGY, pooled analysis of case–control studies on the joint effects of occupational carcinogens in the development of lung cancer; TWA, time-weighted average

## Additional file

Additional file 1: Shows all results not displayed in the main tables in more detail. (DOCX 142 kb)
