# Supplementary material for: Occupational prestige, social mobility and the association with lung cancer in men
Source: BMC Cancer. 2016 Jul 7;16:395. doi: 10.1186/s12885-016-2432-9 (PMC4936282; doi:10.1186/s12885-016-2432-9)
Supplement: Additional file 1: — Shows all results not displayed in the main tables in more detail. (DOCX 142 kb) [file 12885_2016_2432_MOESM1_ESM.docx]

**Supplemental Material**

**Table S1:**  Description of the studies included in the pooled analysis

| **Study** | **Recruitment period** | **Cases**  **N (%)** | **Controls**  **N (%)** | **Control type** |
| --- | --- | --- | --- | --- |
| HdA (Germany) | 1988-1993 | 725 (6.3) | 674 (4.8) | Population |
| AUT (Germany) | 1990-1995 | 2,476 (21.7) | 2,540 (18.0) | Population |
| LUCAS (Sweden) | 1985-1990 | 975 (8.5) | 2,206 (15.6) | Population |
| INCO - Cz Rep. | 1999-2002 | 216 (1.9) | 280 (2.0) | Hospital |
| INCO - Hungary | 1998-2001 | 295 (2.6) | 236 (1.7) | Hospital |
| INCO - Poland | 1998-2002 | 479 (4.2) | 513 (3.6) | Population/hospital |
| INCO - Romania | 1998-2002 | 138 (1.2) | 145 (1.0) | Hospital |
| INCO - Russia | 1998-2001 | 518 (4.5) | 498 (3.5) | Hospital |
| INCO - Slovakia | 1998-2002 | 279 (2.4) | 231 (1.6) | Hospital |
| INCO - UK | 1998-2005 | 263 (2.3) | 543 (3.8) | Population |
| LUCA (France) | 1989-1992 | 237 (2.1) | 244 (1.7) | Hospital |
| TURIN (Italy) | 1990-1994 | 541 (4.7) | 743 (5.3) | Population |
| ROME (Italy) | 1993-1996 | 233 (2.0) | 224 (1.6) | Hospital |
| MONTREAL (Canada) | 1996-1999 | 473 (4.1) | 631 (4.5) | Population |
| EAGLE (Italy) | 2002-2005 | 1,382 (12.1) | 1,510 (10.7) | Population |
| ICARE (France) | 2001-2007 | 1,650 (14.4) | 2,321 (16.4) | Population |
| TORONTO (Canada) | 1996-2002 | 131 (1.1) | 235 (1.7) | Population/hospital |
| CAPUA (Spain) | 2000-2010 | 422 (3.7) | 373 (2.6) | Hospital |
| **TOTAL** |  | **11,433 (100)** | **14,147 (100)** |  |

**Table S2:** Estimated lung cancer risks (OR) with 95% confidence intervals (CI) for occupational social prestige categories of first and last job prestige for whole study population

| **Group** | **Occupational prestige ^a^** | **Cases [N]** | **Controls [N]** | **OR1 (95% CI)** | **OR2 (95% CI)** | **OR3 (95% CI)** | **OR4 (95% CI)** |
| --- | --- | --- | --- | --- | --- | --- | --- |
| First job | High | 1,400 | 2,787 | 1.0 | 1.0 | 1.0 | 1.0 |
|  | Medium | 4,145 | 5,190 | 1.46 (1.36-1.58) | 1.24 (1.14-1.36) | 1.22 (1.11-1.33) | 1.01 (0.88-1.16) |
|  | Low | 5,888 | 6,170 | 1.81 (1.68-1.96) | 1.40 (1.28-1.52) | 1.35 (1.24-1.47) | 1.06 (0.92-1.23) |
|  |  |  |  |  |  |  |  |
| Last job | High | 2,883 | 5,565 | 1.0 | 1.0 | 1.0 | 1.0 |
|  | Medium | 3,036 | 3,502 | 1.64 (1.53-1.76) | 1.44 (1.34-1.56) | 1.42 (1.32-1.53) | 1.15 (1.04-1.28) |
|  | Low | 5,514 | 5,080 | 2.10 (1.98-2.23) | 1.65 (1.54-1.77) | 1.60 (1.50-1.72) | 1.39 (1.25-1.55) |

a Categories for social prestige categories: low (13-≤35 points), medium (>35-≤45 points), high (>45-78 points)

OR1 adjusted for study center and log(age).

OR2 additionally adjusted for smoking status with time since quitting (2-7, 8-15, 16-25 or ≥ 26 years before interview/diagnosis, other types of tobacco only, non-smokers), and log(pack-years+1).

OR3 additionally adjusted for ever working in a “List A” occupation.

OR4 additionally adjusted for highest education.

**Table S3:** Estimated lung cancer risks (OR) with 95% confidence intervals (CI) for time-weighted average social prestige categories for whole study population and major histological subtypes of lung cancer: Equidistant categories and equal number of occupations of prestige score categories

| **Occupational social prestige category** | **Cases**  **[N]** | **Controls**  **[N]** | **OR1 (95% CI)** | **OR2 (95% CI)** | **OR3 (95% CI)** | **OR4 (95% CI)** |
| --- | --- | --- | --- | --- | --- | --- |
| Equidistant categories |  |  |  |  |  |  |
| High (57-78 points) | 741 | 1,805 | 1.0 | 1.0 | 1.0 | 1.0 |
| Medium (35-56 points) | 5,966 | 8,092 | 1.75 (1.59-1.92) | 1.41 (1.27-1.57) | 1.38 (1.25-1.54) | 1.16 (1.03-1.30) |
| Low (13-34 points) | 4,726 | 4,250 | 2.70 (2.45-2.97) | 1.85 (1.65-2.06) | 1.77 (1.59-1.98) | 1.39 (1.22-1.58) |
| Equal number of occupations |  |  |  |  |  |  |
| High (46-78 points) | 2,215 | 4,592 | 1.0 | 1.0 | 1.0 | 1.0 |
| Medium (34-45 points) | 5,299 | 6,013 | 1.79 (1.67-1.90) | 1.48 (1.38-1.60) | 1.45 (1.35-1.56) | 1.29 (1.19-1.40) |
| Low (13-33 points) | 3,919 | 3,542 | 2.33 (2.17-2.50) | 1.69 (1.56-1.83) | 1.62 (1.50-1.76) | 1.40 (1.27-1.53) |

OR1 adjusted for study center and log(age).

OR2 additionally adjusted for smoking status with time since quitting (2-7, 8-15, 16-25 or ≥ 26 years before interview/diagnosis, other types of tobacco only, non-smokers), and log(pack-years+1).

OR3 additionally adjusted for ever working in a “List A” occupation.

OR4 additionally adjusted for highest education.

**Table S4:** Odds ratios (OR) with 95% confidence intervals (CI) between lung cancer and categories for occupational classification of job autonomy

| **Social prestige occupational class ^a^** | **Cases** | **Controls** | **OR1 (95% CI)** | **OR2 (95% CI)** | **OR3 (95% CI)** | **OR4 (95% CI)** |
| --- | --- | --- | --- | --- | --- | --- |
| High autonomy, free-lance academics, persons in leading line management | 272 | 659 | 1.0 | 1.0 | 1.0 | 1.0 |
| Autonomous or employed position with limited managerial responsibility, autonomous functional responsibility, university or technical college degree | 1,013 | 2,339 | 0.97 (0.83-1.14) | 0.93 (0.77-1.12) | 0.93 (0.77-1.11) | 0.87 (0.72-1.05) |
| Limited autonomy, supervised work, medium occupational training | 2,057 | 3,184 | 1.48 (1.27-1.72) | 1.19 (1.0-1.42) | 1.18 (0.99-1.41) | 1.02 (0.85-1.23) |
| Low autonomy, simple activities, mainly in production, services, metal production, traditional handicraft | 4,236 | 4,466 | 2.14 (1.84-2.48) | 1.63 (1.37-1.93) | 1.58 (1.33-1.87) | 1.29 (1.07-1.55) |
| Very low autonomy, manual and unskilled work | 3,855 | 3,499 | 2.57 (2.21-2.99) | 1.70 (1.44-2.02) | 1.63 (1.38-1.94) | 1.29 (1.07-1.56) |

a Categories for social prestige occupational class according to Hoffmeyer-Zlotnik et al. 2003 [23]: Very low autonomy = 13-<33 points, Low autonomy = 33-<42, Limited autonomy = 42-<51, Autonomous or employed position = 51-<64, High autonomy = ≥64 points.

OR1 adjusted for study center and log(age).

OR2 additionally adjusted for smoking status with time since quitting (2-7, 8-15, 16-25 or ≥ 26 years before interview/diagnosis, other types of tobacco only, non-smokers), and log(pack-years+1).

OR3 additionally adjusted for ever working in a “List A” occupation.

OR4 additionally adjusted for highest education

**Table S5:** Further analyses of subpopulations (estimates based on time-weighted average occupational social prestige in control distribution)

| **Subpopulation** | **Occupational prestige** | **Cases [N]** | **Controls [N]** | **OR (95% CI)** |
| --- | --- | --- | --- | --- |
|  |  |  |  |  |
| **Control type** |  |  |  |  |
| Population | High | 1,802 | 3,956 | 1.0 |
|  | Medium | 3,143 | 3,969 | 1.30 (1.18-1.42) |
|  | Low | 4,150 | 3,627 | 1.47 (1.32-1.63) |
| Hospital | High | 573 | 636 | 1.0 |
|  | Medium | 1,041 | 885 | 1.19 (1.0-1.43) |
|  | Low | 1,334 | 1,074 | 1.13 (0.92-1.39) |
| **Excluded study regions** |  |  |  |  |
| Without Northern Europe | High | 971 | 1,592 | 1.0 |
|  | Medium | 1,774 | 1,955 | 1.17 (1.03-1.34) |
|  | Low | 2,362 | 2,072 | 1.23 (1.06-1.41) |
| Without East Europe | High | 1,838 | 4,039 | 1.0 |
|  | Medium | 3,303 | 4,226 | 1.20 (1.10-1.32) |
|  | Low | 4,367 | 3,979 | 1.47 (1.33-1.62) |
| Without Southern Europe | High | 1,763 | 3,882 | 1.0 |
|  | Medium | 3,037 | 3,750 | 1.27 (1.15-1.39) |
|  | Low | 4,055 | 3,665 | 1.52 (1.38-1.67) |
| Without Canada | High | 2,073 | 4,263 | 1.0 |
|  | Medium | 3,826 | 4,631 | 1.22 (1.12-1.33) |
|  | Low | 4,930 | 4,387 | 1.43 (1.31-1.57) |

ORs adjusted for study center, log(age), smoking status with time since quitting (2-7, 8-15, 16-25 or ≥ 26 years before interview/diagnosis, other types of tobacco only, non-smokers), and log(pack-years+1), ever working in a “List A” occupation, and highest school education

**Table S6**: Estimated lung cancer risks (OR) with 95% confidence intervals (CI): Confounder analysis

| **Confounder** | **OR1 (95% CI)** | **p-value** | **OR2 (95% CI)** | **p-value** | **OR3 (95% CI)** | **p-value** | **OR4 (95% CI)** | **p-value** |
| --- | --- | --- | --- | --- | --- | --- | --- | --- |
| **Age[log]** | 1.79 (1.51-2.13) | <0.0001 | 2.25 (1.82-2.78) | <0.0001 | 2.25 (1.82-2.78) | <0.0001 | 2.13 (1.72-2.64) | <0.0001 |
| **Time since quitting** |  |  |  |  |  |  |  |  |
| Never | - |  | 1.0 |  | 1.0 |  | 1.0 |  |
| Ever other types of tobacco only | - |  | 1.35 (1.08-1.68) | 0.0079 | 1.34 (1.07-1.67) | <0.0001 | 1.34 (1.07-1.67) | <0.0001 |
| 2-7 years | - |  | 1.03 (0.82-1.29) | 0.822 | 1.02 (0.81-1.28) | 0.882 | 1.03 (0.52-1.29) | 0.828 |
| 8-15 years | - |  | 0.67 (0.53-0.83) | 0.0004 | 0.66 (0.53-0.83) | 0.0003 | 0.67 (0.53-0.83) | 0.0004 |
| 16-25 years | - |  | 0.53 (0.43-0.66) | <0.0001 | 0.53 (0.43-0.65) | <0.0001 | 0.53 (0.43-0.66) | <0.0001 |
| ≥ 26 years | - |  | 0.51 (0.42-0.63) | <0.0001 | 0.51 (0.42-0.62) | <0.0001 | 0.51 (0.42-0.63) | <0.0001 |
| Current | - |  | 5.88 (4.65-7.43) | <0.0001 | 5.88 (4.66-7.44) | <0.0001 | 5.94 (4.70-7.51) | <0.0001 |
| **Pack-years+1 [log]** | - |  | 2.18 (2.08-2.29) | <0.0001 | 2.18 (2.08-2.30) | <0.0001 | 2.17 (2.07-2.28) | <0.0001 |
| **List A Job** |  |  |  |  |  |  |  |  |
| Never | - |  | - |  | 1.0 |  | 1.0 |  |
| Ever | - |  | - |  | 1.31 (1.20-1.43) | <0.0001 | 1.29 (1.18-1.41) | <0.0001 |
| **Education** |  |  |  |  |  |  |  |  |
| University | - |  | - |  | - |  | 1.0 |  |
| 10-13 years | - |  | - |  | - |  | 1.20 (1.05-1.38) | <0.0111 |
| 6- <10 years | - |  | - |  | - |  | 1.32 (1.16-1.50) | <0.0001 |
| <6 years | - |  | - |  | - |  | 1.57 (1.12-2.20) | <0.0001 |

OR1 adjusted for study center and log(age).

OR2 additionally adjusted for smoking status with time since quitting (2-7, 8-15, 16-25 or ≥ 26 years before interview/diagnosis, other types of tobacco only, non-smokers), and log(pack-years+1).

OR3 additionally adjusted for ever working in a “List A” occupation.

OR4 additionally adjusted for highest education.

***
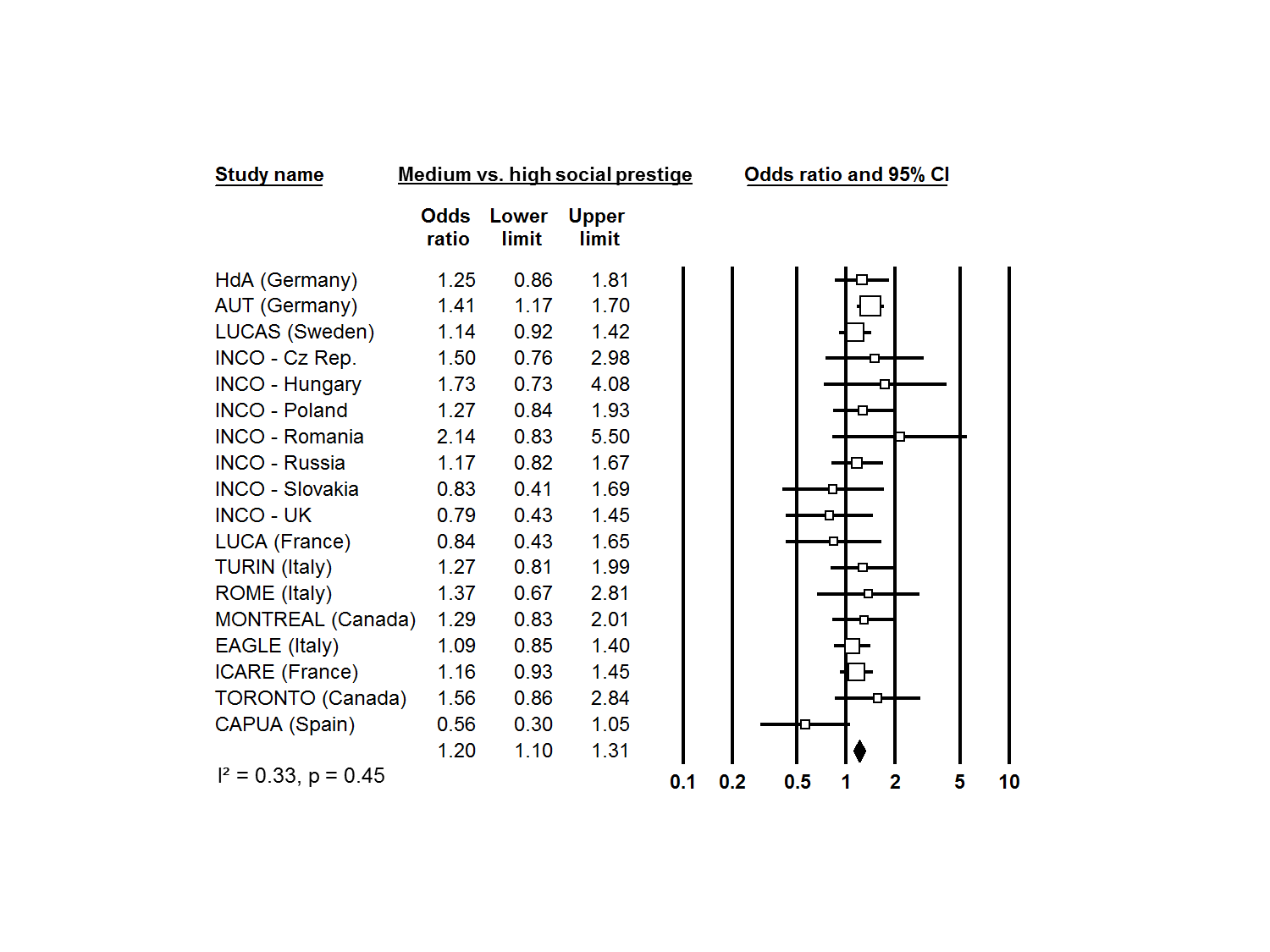

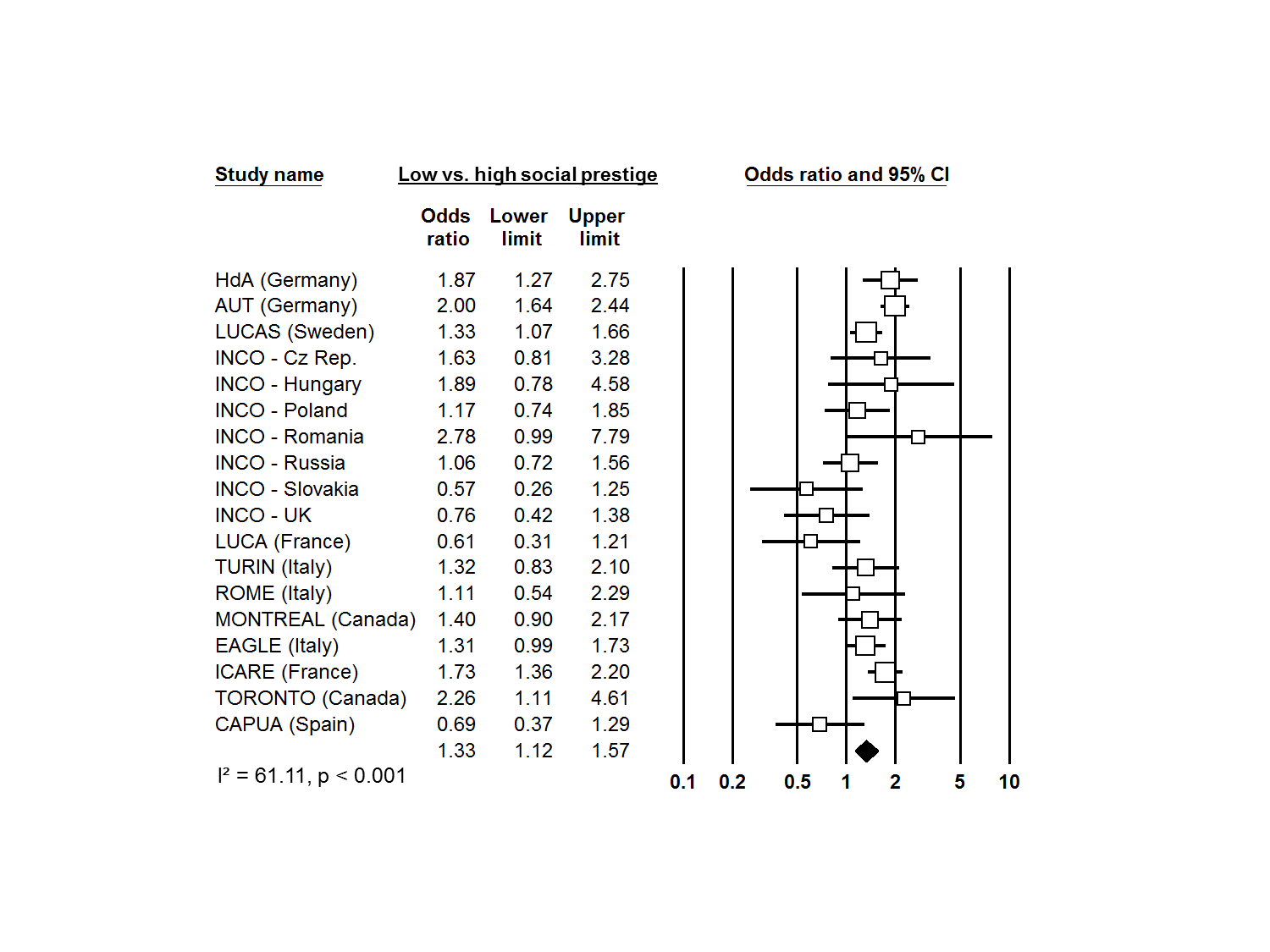
***

**Figure S1**: Forest plot of risk estimates by center: (A) Medium vs. high occupational social prestige, (B) Low vs. high prestige (estimates based on tertiles of time-weighted average prestige in controls. Risk estimates are fully adjusted, including education (model 4).

**Figure S2** Unadjusted time course of mean occupational social prestige with 95% confidence intervals for working durations from 0 to 50 years (by intervals of 5 years) for cases and controls (class limits based on tertiles of the distribution of TWA-prestige among controls): Subjects starting with high prestige

**Figure S3** Unadjusted time course of mean occupational social prestige with 95% confidence intervals for working durations from 0 to 50 years (by intervals of 5 years) for cases and controls (class limits based on tertiles of the distribution of TWA-prestige among controls): Subjects starting with medium prestige

**Fig. S4** Unadjusted time course of mean occupational social prestige with 95% confidence intervals for working durations from 0 to 50 years (by intervals of 5 years) for cases and controls (class limits based on tertiles of the distribution of TWA-prestige among controls): Subjects starting with low prestige

**Figure S5:** Unadjusted time course of mean social prestige with 95% confidence intervals for age (by intervals of 5 years) for cases and controls (class limits based on tertiles of the distribution of TWA average occupational social prestige in controls):

Subjects starting with high prestige

**Figure S6:** Unadjusted time course of mean social prestige with 95% confidence intervals for age (by intervals of 5 years) for cases and controls (class limits based on tertiles of the distribution of TWA average occupational social prestige in controls):

Subjects starting with medium prestige

**Figure S7:** Unadjusted time course of mean social prestige with 95% confidence intervals for age (by intervals of 5 years) for cases and controls (class limits based on tertiles of the distribution of TWA average occupational social prestige in controls):

Subjects starting with low prestige
